# Methyltransferase‐Like 3‐Mediated N^6^ ‐Methyladenosine Modification on RNAs: A Novel Perspective for the Pathogenesis and Treatment of Bone Diseases

**DOI:** 10.1111/jcmm.70483

**Published:** 2025-03-07

**Authors:** Dongqiong Xiao, Deshuang Zhang, Yi Qu, Xiaojuan Su

**Affiliations:** ^1^ Department of Pediatrics, Key Laboratory of Birth Defects and Related Diseases of Women and Children (Ministry of Education), NHC Key Laboratory of Chronobiology West China Second University Hospital, Sichuan University Chengdu Sichuan China; ^2^ Division of Neonatology, Department of Pediatrics The Affiliated Hospital of Southwest Medical University Luzhou Sichuan China

**Keywords:** diagnosis, methyltransferase‐like 3, osteoarthritis, osteoporosis, osteosarcoma, RNA N^6^‐methyladenosine modification, treatment

## Abstract

Osteoarthritis, osteoporosis, and osteosarcoma are prevalent osseous pathologies associated with the aberrant functionality of chondrocytes, osteoclasts, and osteoblasts, respectively. These conditions frequently exhibit therapeutic resistance and possess a high mortality risk, thus representing substantial health threats. To mitigate these concerns, it is imperative to investigate novel mechanistic insights. Methyltransferase‐like 3 (METTL3) is pivotal in these disorders by modulating gene expression via N^6^‐methyladenosine (m^6^A) modifications on RNA, thereby impacting cellular processes. Although considerable research has elucidated METTL3's involvement in these diseases, a systematic review is essential to summarise these findings and evaluate METTL3's significance. This review endeavours to aggregate and examine contemporary studies to elucidate METTL3's role in bone pathologies and its clinical implications. We propose that METTL3 constitutes a risk gene in these conditions by mediating m^6^A modifications on both mRNAs and non‐coding RNAs, suggesting that METTL3 may serve as a critical diagnostic biomarker and therapeutic target. In conclusion, this review provides an extensive analysis of METTL3 and its correlation with osteoarthritis, osteoporosis, and osteosarcoma, offering valuable perspectives on extant research and serving as a valuable reference for researchers engaged in both basic and translational studies.

## 1 Introduction

Osteoclasts, osteoblasts, and chondrocytes are the primary cellular components responsible for bone formation and determining bone functionality. The malfunction of these cell types is implicated in various bone pathologies, notably osteoarthritis (OA), osteoporosis (OP), and osteosarcoma (OS) [1]. These conditions are characterised by high prevalence rates, frequent treatment failures, and significant mortality, thereby adversely affecting the quality of life for individuals. Nevertheless, the underlying mechanisms of pathogenesis remain incompletely understood. Thus, it is imperative to investigate this issue from novel perspectives, which may yield beneficial insights.

Genetic factors are widely acknowledged as fundamental contributors to a range of human diseases, including OA, OP, and OS. Gene expression can be modulated at both transcriptional and post‐transcriptional levels [2, 3]. One of the most significant forms of post‐transcriptional gene regulation is N^6^‐methyladenosine (m^6^A) modification, which affects messenger RNAs (mRNAs) and non‐coding RNAs within cells [4, 5, 6]. The impact of m^6^A modification on RNA is dependent on the interplay among three classes of regulatory proteins: “writers” (primarily the methyltransferase complex consisting of methyltransferase‐like 3/14 [METTL3/METTL14] and WTAP), “erasers,” and “readers” [7]. Specifically, writers facilitate the addition of m^6^A marks, erasers remove them, and readers recognise these modifications, thereby influencing RNA splicing, translation, nucleosynthesis, and degradation, which ultimately shape gene expression and cellular functions [8, 9, 10]. Notably, the mechanism of RNA m^6^A modification is contingent upon the interactions among m^6^A regulatory proteins. METTL3, a quintessential methyltransferase among m^6^A regulators, acts as a functional subunit in RNA m^6^A modification, operating both dependently and independently of its catalytic activity. Conversely, METTL14 predominantly functions as a structural support subunit for m^6^A [8, 9].

To date, extensive research has elucidated the roles and mechanisms of METTL3 in OA, OP, and OS. However, a thorough review that interprets the interconnections among these diseases and proposes potential clinical applications for METTL3 is currently absent. This review aims to synthesise and critically analyse the literature regarding METTL3's involvement in OA, OP, and OS, alongside its molecular mechanisms. Additionally, we propose clinical applications of METTL3 within this framework and underscore existing challenges in comprehending METTL3's role in these pathologies.

## 2 Mechanistic Insights Into the Role of METTL3 in Osteopathies

### 2.1 METTL3 Contributes to OA Onset and Progression

OA, the most prevalent type of rheumatoid arthritis, involves the degeneration of joint cartilage throughout the body [11]. Chondrocytes, the primary cells in cartilage, play a crucial role in maintaining cartilage stability by producing and breaking down the extracellular matrix (ECM) [12]. Various proteases, such as matrix metalloproteinases (MMPs), are involved in ECM degradation. Notably, type II collagen (Coll II) is a significant component of cartilage ECM, and research indicates that MMP1, MMP3, and MMP13 contribute to its degradation by reducing Coll II levels [13]. Additionally, tissue inhibitors of metalloproteinases (TIMPs) regulate MMP activity, and the balance between TIMPs and MMPs is vital for the normal functioning of OA [14]. Furthermore, METTL3 is implicated in the dysregulation of chondrocytes and ECM degradation, which leads to the development and progression of OA.

#### 2.1.1 METTL3 Functions by Targeting mRNAs

##### 2.1.1.1 METTL3 Triggers Inflammation

**FIGURE 1:**
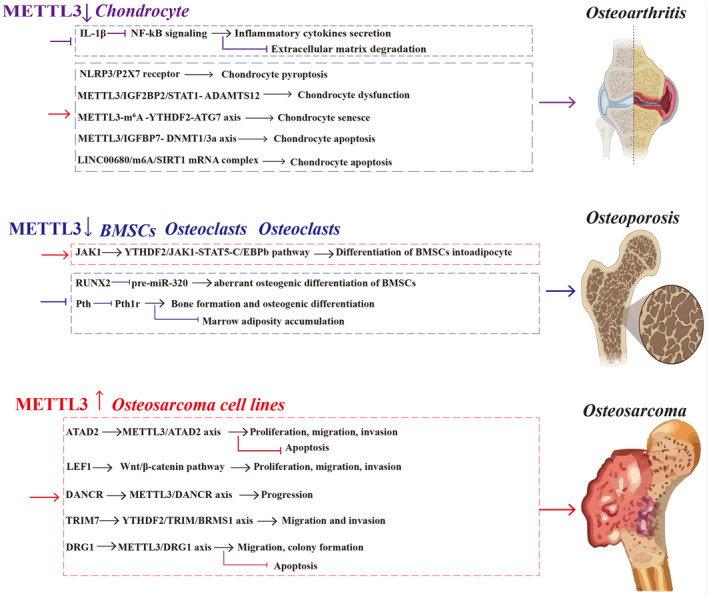
The effect of METTL3 on crucial pathways influencing bone diseases. METTL3 exhibits aberrant expression levels in conditions such as osteoarthritis, osteoporosis, and osteosarcoma, functioning through diverse mechanisms by targeting RNAs in distinct cellular contexts. A thorough classification and comprehensive summary of METTL3's targets, mechanisms, and roles across various cell types and their associated bone pathologies are provided. METTL3, methyltransferase‐like 3. m^6^A, N^6^‐methyladenosine. BMSCs, bone marrow‐derived mesenchymal stem cells. NLRP3, nod‐like receptor pyrin domain 3 inflammasomes. ADAMTS12, A disintegrin and metalloproteinase with thrombospondin motifs 12. STAT1, signal transducer and activator of transcription 1. FLS, fibroblast‐like synoviocyte. ATG7, autophagy‐related. IGFBP7, lncRNA IGFBP7‐OT. LINC00680, lncRNA LINC00680. Pth, parathyroid hormone. Pth1r, parathyroid hormone 1 receptor. RUNX2, runt‐related transcription factor 2. JAK1, janus kinase 1.LEF1, lymphoid enhancer binding factor 1. TRIM7, tripartite motif 7. DRG1, developmentally regulated GTP‐binding protein 1.

Pro‐inflammatory factors such as IL‐1β, IL‐18, and TNF‐α accelerate cartilage dysfunction and OA progression by increasing the death and functions of chondrocytes in ECM degradation [15]. The elevated level of IL‐1β triggers inflammatory cascade reactions in chondrocytes and is involved in OA development by serving as the inducer of cartilage degeneration [16]. IL‐1β upregulates METTL3 expression in chondrocytes in a dose‐dependent manner [17]. A high level of METTL3 increases IL‐1β‐induced apoptosis, inflammatory cytokine production, and NF‐kB signalling activation in chondrocytes. Moreover, METTL3 promotes ECM degradation by inducing the expression of MMP13 and Coll X, suppressing the expression of aggrecan and Coll II [17]. Therefore, METTL3 contributes to the onset and progression of OA by regulating ECM synthesis in chondrocytes via the NF‐κB signalling. Collectively, METTL3 contributes to OA progression probably by regulating the inflammatory response, which in turn affects ECM degradation by adjusting the balance between TIMPs and MMPs (Figure 1, Table 1).

**TABLE 1:** Impairment of METTL3 function facilitates the initiation and advancement of OA.

Risk factors for OA	METTL3			Reference
	Target	Regulate	Function	
IL‐1β	*MMP13 and Coll X*	Up	ECM degradation	[17]
	*Aggrecan and Coll II*	Down		
P2X7 receptor	*NLRP3*	Up	Chondrocyte pyroptosis	[18]
ADAMTS12	*STAT1*	Up	Chondrocyte dysfunction	[20]
FLS	*ATG7*	Down	Chondrocyte senesce	[22, 23]
IGFBP7	*DNMT1/3a*	Down	OA progression	[24]
LINC00680	*SIRT1*	Up	OA progression	[25]

*Note:* Risk factors lead to METTL3 dysfunction in OA, which then mediates m^6^A modifications on various target RNAs, affecting their translation and expression. This ultimately alters cell functions and contributes to the development and progression of OA.

Abbreviations: ADAMTS12, A disintegrin and metalloproteinase with thrombospondin motifs 12; ATG7, autophagy‐related; Coll II, type II collagen; ECM, extracellular matrix; FLS, fibroblast‐like synoviocyte; IGFBP7, lncRNA IGFBP7‐OT; IL, interleukin; LINC00680, lncRNA LINC00680; m^6^A, N^6^‐methyladenosine; METTL3, methyltransferase‐like 3; MMP, metalloproteinases; NLRP3, nod‐like receptor pyrin domain 3 inflammasomes; OA, osteoarthritis; STAT1, signal transducer and activator of transcription 1.

##### 2.1.1.2 METTL3 Triggers Chondrocyte Pyroptosis and Dysfunction

Pyroptosis is a kind of programmed cell death caused by a strong inflammatory response. Li et al. [18] found that the METTL3/NF‐*κ*B/nod‐like receptor pyrin domain 3 inflammasomes (NLRP3) crosstalk contributes to the inflammatory response in P2X7 receptor‐induced chondrocyte pyroptosis, leading to cartilage degradation and OA progression. Furthermore, A disintegrin and metalloproteinase with thrombospondin motifs 12 (ADAMTS12) is a cartilage oligomeric matrix protein‐cleaving enzyme, which controls the structure and function of ECM [19]. The aberrant expression or function of ADAMTS12 contributes to OA. Yang et al. [20] found that METTL3/IGF2BP2‐mediated m^6^A modification on signal transducer and activator of transcription 1 (*STAT1*) promotes *STAT1* mRNA stability and increases its expression, which subsequently binds to the promoter region of ADAMTS12 to activate its transcription and expression. Consistently, the upregulated expression of ADAMTS12 contributes to IL‐1β‐induced chondrocyte dysfunction and OA progression [20]. These findings collectively suggest that the METTL3/IGF2BP2‐mediated STAT1 methylation promotes OA progression by up‐regulating ADAMTS12 expression. Therefore, METTL3 acts as the key regulator for the aberrant expression of ADAMTS12, targeting which might be therapeutic for OA (Figure 1, Table 1).

##### 2.1.1.3 METTL3 Triggers Chondrocyte Senesce

An increase in the number of senescent chondrocytes is one important pathological feature for patients with OA [21]. Senescent chondrocytes are unable to divide, resistant to apoptosis, and robust enough to secrete proinflammatory cytokines and MMPs [21]. The mechanism for chondrocyte senescence is associated with METTL3‐mediated m^6^A RNA modification. During OA progression, Chen et al. [22] found that the senescent fibroblast‐like synoviocyte (FLS) is markedly increased, which induces chondrocyte senescence and cartilage degradation by secreting proinflammatory cytokines and MMPs. Conversely, METTL3 suppression in FLS effectively inhibits chondrocyte senescence and attenuates OA progression in the OA mouse model. METTL3‐mediated m^6^A modification decreases the expression of autophagy‐related (ATG) 7 by affecting the *ATG7* mRNA stability in an m^6^A‐YTHDF2‐dependent manner, which is crucial for autophagosome formation and autophagy activity [22]. Therefore, METTL3 is the key regulator of chondrocyte senescence during OA progression, which functions by targeting the METTL3‐m^6^A‐YTHDF2‐ATG7 axis to regulate chondrocyte autophagy. Furthermore, mechanical overloading triggers chondrocyte senescence and induces OA progression that is regulated by YAP. Yang et al. [23] reported that the mechanism for YAP‐induced chondrocyte senescence is related to the enhancement of METTL3‐mediated m^6^A modification [23]. Inhibition of METTL3 rescues chondrocyte senescence and suppresses OA progression [23]. These studies shed insight into developing preventive and curative strategies for OA by targeting METTL3 and METTL3‐methylated mRNA (Figure 1, Table 1).

#### 2.1.2 METTL3 Functions by Targeting Non‐Coding RNAs

In addition to functions in OA by regulating mRNAs, METTL3‐mediated m^6^A modification on lncRNAs is also involved in the onset and progression of OA, which depends on its effect on cartilage tissue destruction, inflammatory events, autophagy, and apoptosis. For example, LncRNA IGFBP7‐OT (IGFBP7) is upregulated in OA and promotes OA progression, which is most likely linked to the METTL3‐mediated m^6^A modification on *IGFBP7* that in turn reduces the binding of DNMT1/3a to the IGFBP7 promoter, thereby decreasing DNA methylation of the IGFBP7 promoter, and consequently upregulating IGFBP7 expression [24]. Besides, Ren et al. [25] demonstrated that METTL3‐mediated m^6^A modification on lncRNA LINC00680 increases LINC00680 levels in OA tissue and IL‐1β‐induced chondrocytes, which interacts with *SIRT1* mRNA and enhances *SIRT1* mRNA stability. Overall, these findings show the role of the LINC00680/m^6^A/*SIRT1* mRNA complex in chondrocytes and uncover the mechanism by which METTL3‐mediated LINC00680 accelerates OA progression, which may provide novel insight for understanding the underlying mechanism for OA pathogenesis (Figure 1, Table 1).

In summary, these studies suggest that high levels of METTL3 are a risk factor for OA, which functions by mediating m^6^A modification on both mRNAs and non‐coding RNAs, thereby triggering the inflammation response, accelerating ECM degradation, regulating chondrocyte death such as apoptosis, autophagy, ferroptosis, and pyroptosis, as well as promoting chondrocyte dysfunction.

### 2.2 METTL3 Contributes to OP Pathogenesis

Human OP is a common systematic skeletal disease that is characterised by enhanced bone fragility and often results in bone fractures, which is prevalent in older populations and postmenopausal women [26]. Bone metabolism, bone mass, bone microstructure, bone mineral density, and bone tissue degradation are prominent symptoms of OP that are regulated by various bone cells [26]. METTL3 dysregulation contributes to residual ridge resorption dysfunction [27].

#### 2.2.1 METTL3 Functions by Mediating m^6^A Modification on mRNAs

Wang et al. [28] found that EGR1 promotes *METTL3* transcription and increases m^6^A‐modified *CHI3L1* mRNA level, thereby stimulating the generation of osteoclasts and OP development through the METTL3/m^6^A/CHI3L1 axis. These findings suggest that *METTL3* dysregulation plays a crucial role in OP pathogenesis by regulating bone metabolism. Besides, Wu et al. [29] reported that suppression of METTL3 activity in BMSCs results in impaired bone formation. Conversely, activation of METTL3 expression in BMSCs is protective for OP mice. These findings suggest that the activated role or a high level of METTL3 is critical for the function of BMSCs in bone formation and bone mass. In line with this study, another study demonstrated that deletion of METTL3 impairs BMSC differentiation into osteoblasts while enhancing BMSC differentiation into adipocytes, which impairs bone microstructure and bone mineral density, promoting bone tissue degradation and enhancing marrow adiposity, indicating that the role of METTL3 in regulating BMSC differentiation controls bone fates, dysfunction of which is a risk factor for OP [29]. METTL3 functions by impeding the translation of parathyroid hormone (Pth)/parathyroid hormone 1 receptor (Pth1r) signalling axis, a lineage allocator for BMSCs [29]. Taken together, a low level of METTL3 enhances the differentiation of BMSCs into adipocytes and suppresses osteoblast generation by targeting the Pth/Pth1r signalling axis, which is a mechanism for OP pathogenesis. Moreover, Yao et al. [30] discovered that METTL3 knockdown in porcine BMSCs not only facilitates adipogenesis but also enhances the expression of the Janus kinase 1 (JAK1) protein, which functions by increasing *JAK1* mRNA stability in a YTHDF2‐dependent manner. This subsequently activates the STAT5 through phosphorylation and binds to the promoter of CCAAT/enhancer binding protein (*C*/*EBP*) [30]. Therefore, METTL3 plays a pivotal role in promoting the differentiation of BMSCs into adipocytes by targeting the YTHDF2‐JAK1‐STAT5‐C/EBPb pathway (Figure 1, Table 2).

**TABLE 2:** Inhibition of METTL3 expression promotes the onset and progression of OP.

METTL3			Reference
Target	Regulate	Function	
*CHI3L1*	Up	Generation of osteoclasts	[28]
*Pth/Pth1r*	Down	Enhance the differentiation from BMSC into adipocytes	[29]
*RUNX2*	Down	Reduce BMSC osteogenic differentiation	[31]
*JAK1*	Up	Enhance the differentiation from BMSC into adipocytes	[30]
*LINC00657*	Down	Reduce BMSC osteogenic differentiation	[32]
*LncRNA MIR99AHG*	Up	Reduce BMSC osteogenic differentiation	[33]

*Note:* In osteoporotic conditions, METTL3 is significantly downregulated, which plays a crucial role by targeting various RNAs to modulate their expression. This regulation ultimately leads to an imbalance in the differentiation of BMSCs between adipocytes and osteogenic cells.

Abbreviations: BMSCs, bone marrow‐derived mesenchymal stem cells; JAK1, janus kinase 1; METTL3, methyltransferase‐like 3; OP, osteoporosis; Pth, parathyroid hormone; Pth1r, parathyroid hormone 1 receptor; RUNX2, runt‐related transcription factor 2.

#### 2.2.2 METTL3 Functions by Mediating m^6^A Modification on Non‐Coding RNAs

Meanwhile, a low expression level of METTL3 inhibits the expression of osteogenic genes (such as *RUNX2*), alkaline phosphatase activity, and the formation of mineralized nodules, and even inhibits PI3K‐Akt signalling, which reduces the osteogenic differentiation ability of BMSCs and leads to a reduction in bone mass in people with OP. This is achieved by augmenting the m^6^A methylation on *RUNX2*, a key transcription factor for osteoblast differentiation and bone formation, as well as pre‐miR‐320 [31]. In summary, a low level of METTL3 facilitates the aberrant osteogenic differentiation of BMSCs through the m^6^A‐mediated direct and indirect regulatory effects of the pre‐miR‐320/RUNX2 axis in OP. Furthermore, Peng et al. [32] found that METTL3 is downregulated in BMSCs of patients with OP, which mediates m^6^A methylation on LINC00657. The methylated‐LINC00657 serves as a competing endogenous RNA to upregulate BMPR1B via sponging miR‐144‐3p, finally promoting OP development. Notably, METTL3 inhibits the osteogenic differentiation of BMSCs via the LINC00657/miR‐144‐3p/BMPR1B axis. Therefore, upregulation of METTL3 or downregulation of BMPR1B alleviates the differentiation imbalance of BMSCs and improves the bone formation ability of patients with OP. Li et al. [33] demonstrated that METTL3‐mediated LncRNA MIR99AHG methylation enhances the osteogenic differentiation of BMSCs via targeting miR‐4660 (Figure 1, Table 2).

In summary, these findings show that a high level of METTL3 maintains the differentiation balance of BMSCs between adipose and osteogenic differentiation. However, suppression or dysfunction of METTL3 reduces osteogenic differentiation and enhances adipogenic differentiation from BMSCs, leading to OP onset and progression. Therefore, METTL3 serves as a key inducer for the pathogenesis of OP, suggesting the potential of METTL3 as a diagnostic biomarker and a therapeutic target.

### 2.3 METTL3 Contributes to the Pathogenesis and Recurrence of OS

Human OS, a primary bone tumour commonly found in children and young adults, is notorious for its high rates of recurrence and metastasis due to the aggressive features of tumour cell migration, invasion, and chemotaxis [34]. Recent studies have identified METTL3 as a significant contributor to the pathogenesis and recurrence of OS [35]. Specifically, studies have shown that the level of METTL3 is significantly upregulated in OS tissues, particularly those associated with metastatic tumours, indicating a strong correlation with poor prognosis [35, 36, 37, 38].

#### 2.3.1 METTL3 Might Be a Novel Oncogene for OS

Elevated levels of METTL3 have been found in both the cytoplasm and nucleus of OS cells, which promote ATAD2 expression that consequently facilitates tumour cell proliferation, migration, and invasion while suppressing apoptosis [39]. In line with this study, other studies report that METTL3 promotes the occurrence and progression of OS by enhancing the stability and expression of *DANCR* mRNA [40], *MALAT1* mRNA [41], *TRAF6* mRNA [42], *COPS5* mRNA [43], and *ZBTB7*C mRNA [44] in OS tissues and cells. These studies suggest that METTL3 functions as an oncogene to methylate other mRNAs in driving OS growth and invasion, thereby presenting as a promising therapeutic target for OS treatment. Jiang et al. [45] found that the upregulated level of METTL3 is correlated with the tumour size, clinical stage, and distant metastasis of patients with OS. Therefore, a high level of METTL3 could be used as a potential diagnostic or prognostic biomarker for patients with OS. The upregulated METTL3 subsequently enhances HDAC5 expression in OS cells by increasing *HDAC5* m^6^A level, which in turn reduces the enrichment of H3K9/K14ac on the miR‐142 promoter, thus suppressing miR‐142‐5p expression and upregulating armadillo‐repeat‐containing 8 (ARMC8) level [45]. Consistently, Wei et al. [46] found that the upregulated level of METTL3 increases the m^6^A level of CCR4‐NOT transcription complex subunit 7 (*CNOT7*) mRNA and CNOT7 expression in a YTHDF1‐dependent manner. Furthermore, Miao et al. [47] uncovered a novel mechanism underlying elevated METTL3 levels in OS, which activates the lymphoid enhancer‐binding factor 1(LEF1)/Wnt/b‐catenin signalling pathway. Taken together, METTL3 acts as the key regulator of the signalling axis of HDAC5‐H3K9/K14ac‐ miR‐142‐ARMC8 and METTL3‐YTHDF1‐CONT7, as well as the LEF1/Wnt/b‐catenin signalling pathway, which promote cell proliferation, migration, and invasion. Therefore, regulating these signalling axes or pathways might be therapeutic for OS (Figure 1, Table 3).

**TABLE 3:** METTL3 contributes to the oncogenesis and advancement of OS.

METTL3					
Disease	Cell lines	Target	Regulate	Function	Reference
*OS*	*SAOS‐2 MG63*	*ATAD2*	Up	Oncogene	[39]
	*Saos‐2*, *SJSA‐1*, *MG63*, *HOS*, *U‐2OS*	*DANCR*	Up		[40]
	*MG‐63*, *U2OS*	*MALAT1*	Up		[41]
	*U2OS*, *MG‐63*, *Saos2*, *HOS*	*TRAF6*	Up		[42]
	*MG63*, *U2OS*	*COPS5*	Up		[43]
	*MNNG/HOS*, *MG63*, *LO2*	*ZBTB7*C	Up		[44]
	*U2OS*, *HOS*, *SAOS2*	*HDAC5*	Up		[45]
	*MG63*, *U2OS*, *HOS*, *Saos‐2*	*CNOT7*	Down		[46]
	*HOS*, *SAOS‐2*	*LEF1*	Up		[47]
	*HOS*, *SAOS2*, *U2‐OS*, *MG63*	*TRIM7*	Up	OS‐risk‐associated genes	[48]
	*Saos‐2*, *U2OS*, *MG63*, *143B*	*DRG1*	Up		[49]
	*143B*, *HOS*	*USP13*	Up		[51]
	*MG63*, *HOS*, *U2OS*, *SAOS2*	*DIRAS1*	Down		[52]
	*G63*, *U2OS*	*circNRIP1*	Up		[53]
	*SaOS‐2*, *HOS*	*circRNF220*	Up		[54]

*Note:* METTL3 is significantly upregulated in the tumorigenesis and progression of OS, acting either directly as an oncogene or indirectly by influencing other genes associated with OS risk.

Abbreviations: CNOT7, CCR4‐NOT transcription complex subunit 7; DIRAS1, DIRAS family GTPase 1; DRG1, developmentally regulated GTP‐binding protein 1; HDAC5, histone deacetylase 5; LEF1, lymphoid enhancer binding factor 1; METTL3, methyltransferase‐like 3; OS, osteosarcoma; TRAF6, TNF receptor‐associated factor 6; TRIM7, tripartite motif 7.

#### 2.3.2 METTL3 Contributes to OS by Targeting Tumour‐Related Genes

Remarkably, in some cases, METTL3 functions to promote OS onset and progression by regulating the expression of OS risk‐associated genes. For example, a heightened level of tripartite motif 7 (TRIM7) is often indicative of poor prognosis in patients with OS [48]. Zhou et al. [48] discovered that by collaborating with YTHDF2, METTL3 reduces the m^6^A modification on *TRIM7* mRNA and enhances TRIM7 expression, which subsequently facilitates the ubiquitination of its target breast cancer metastasis suppressor 1 (*BRMS1*), ultimately promoting the migration and invasion of OS cells. Similarly, the elevated levels of developmentally regulated GTP‐binding protein 1 (*DRG1*) mRNA and protein serve as a hallmark for advanced clinical stages and large tumour sizes in patients with OS [49]. Ling et al. [49] discovered that METTL3 facilitates the stabilisation of *DRG1* mRNA, thereby inducing DRG1 overexpression, which subsequently exerts oncogenic effects, promoting migration and colony formation while inhibiting apoptosis in OS. Moreover, reprogramming metabolism is a hallmark of cancer cells for rapid progression [50]. Specifically, the deubiquitinating enzyme USP13 is found to be upregulated in OS specimens and promotes OS progression through regulating aerobic glycolytic reprogramming [51]. Wang et al. [51] demonstrated that USP13 takes *METTL3* as a target, which promotes glycolysis and tumour progression in OS by stabilising *METTL3*, thereby stabilising *ATG5* mRNA and facilitating autophagy in OS. These findings suggest that USP13 acts as an oncogene and regulates glycolytic reprogramming and progression in OS by stabilising the METTL3/m^6^A/ATG5 axis. In contrast, DIRAS family GTPase 1 (*DIRAS1*) is a tumour suppressor gene and locates in the nucleus of OS cells. Liu et al. [52] found that METTL3‐mediated m^6^A modification on *DIRAS1* mRNA subsequently inhibits DIRAS1 expression in OS cells, which promotes proliferation, invasion, and migration abilities, as well as blocks the apoptosis ability by inhibiting the ERK pathway.

Consistently, METTL3‐mediated m^6^A modification on circRNA is associated with the occurrence and progression of OS. Meng et al. [53] indicated that METTL3‐mediated m^6^A modification on circNRIP1 enhances circNRIP1 expression in OS, which subsequently induces FOXC2 expression by sponging to miR‐199a and promotes OS progression. Therefore, METTL3‐induced circNRIP1 overexpression exerts an oncogenic role in OS by sponging to miR‐199a, providing new ideas for OS treatment. Meanwhile, METTL3‐modulated circRNF220 acts as a sponge for miR‐330‐5p and promotes OS progression by upregulating survivin expression [54] (Figure 1, Table 3).

Current available studies indicate that a high level of METTL3 plays oncogenic roles in the pathogenesis and recurrence of OS. Specifically, the abnormally upregulated METTL3 commonly promotes tumour proliferation, migration, and invasion while suppressing apoptosis, indicating a strong correlation of METTL3 with poor prognosis. Therefore, these studies infer that METTL3 is a potential biomarker for diagnosis or prognosis for people with OS. Further, downregulating METTL3 might be a therapeutic target for patients with OS, which needs further exploration and validation.

## 3 Modulating METTL3 as a Therapeutic Strategy for Managing Osteopathies

### 3.1 Targeting METTL3 Is Effective for OA Treatment

METTL3 overexpression triggers inflammation, accelerates ECM degradation, and promotes various forms of cell death, such as apoptosis, autophagy, ferroptosis, and pyroptosis, all of which contribute to the onset and progression of OA [55]. Thus, managing inflammation could be a crucial approach for treating OA.

#### 3.1.1 Inhibition Of METTL3 Mitigates Inflammatory Responses

Given that the overexpression of METTL3 exacerbates chondrocyte inflammation, cellular apoptosis, and the onset of OA, the downregulation of METTL3 emerges as a viable strategy for managing inflammation and treating OA. Notably, ADAMTS12 has been implicated in the progression of OA. Research indicates that the silencing of METTL3, both in vivo and in vitro, leads to a reduction in ADAMTS12 expression and diminishes IL‐1β‐induced inflammation in chondrocyte injury and cartilage tissues, thereby alleviating OA [20]. Furthermore, Shi et al. demonstrated that the depletion of ribosomal protein L38 (RPL38) inhibits IL‐1β‐triggered apoptosis, inflammation, and ECM degradation in chondrocytes, thus mitigating chondrocyte dysfunction and the advancement of OA. RPL38 represses SOCS2 expression through METTL3‐mediated m^6^A modification of SOCS2, subsequently obstructing the SOCS2‐mediated JAK2/STAT3 signalling pathway [56] (Figure 1, Table 4).

**TABLE 4:** Downregulating METTL3 is effective for OA treatment.

Treatment	METTL3			Reference
	Target	Regulate	Function	
METTL3	*ADAMTS12*	Down	Inhibit inflammatory	[20]
RPL38	*SOCS2*	Down	Anti‐apoptosis, inflammation, ECM degradation	[56]
CREB	*TFEB*	Up	Autophagy activity	[57]
Morroniside	*MMP13, Caspase‐1, NLRP3*	Down	Anti‐apoptosis	[58]
NEK7	*DNMT1/3a*	Up	Anti‐pyroptosis	[59]
BMSC‐EVs	*ACSL4*	Up	Anti‐ferroptosis	[60]
hucMSCs	*NLRP3*	Down	Anti‐apoptosis, pyroptosis	[61]

*Note:* METTL3 exhibits aberrant overexpression in OA. The therapeutic modulation of METTL3 through specific intervention strategies is beneficial for OA treatment, as it operates by targeting various RNAs to modulate chondrocyte apoptosis.

Abbreviations: ADAMTS12, A disintegrin and metalloproteinase with thrombospondin motifs 12; BMSC, bone mesenchymal stem cell; CREB, element binding protein; ECM, extracellular matrix; EVs, extracellular vehicles; hucMSCs, human umbilical cord mesenchymal stem cells; METTL3, methyltransferase‐like 3; MMP, metalloproteinases; NEK7, NIMA‐related kinase 7; NLRP3, nod‐like receptor pyrin domain 3 inflammasomes; OA, osteoarthritis; RPL38, ribosomal protein L38.

#### 3.1.2 Targeting METTL3 Rescues Chondrocyte Death

Dysregulated autophagy leading to cell death is a significant factor in OA. The cAMP response element‐binding protein (CREB) plays a protective role against this dysregulation. In chondrocytes treated with tert‐butyl hydroperoxide, Zhang et al. [57] demonstrated that CREB treatment mitigates autophagy blockage by activating miR‐373 expression. MiR‐373 enhances chondrocyte autophagy by downregulating METTL3 and promoting the release of the autophagy‐related gene TFEB from METTL3. By modulating miR‐373 levels, which directly target METTL3, CREB reduces the m6A suppression of TFEB mediated by METTL3, thereby alleviating OA damage. Thus, CREB's therapeutic action in OA is linked to the miR‐373/METTL3/TFEB pathway, with METTL3 as the key regulator. Similarly, Yu et al. [58] found that Morningside treatment lowers the expression of MMP13, Caspase‐1, and NLRP3 in OA mice and IL‐1β‐stimulated chondrocytes. Morningside also slows OA progression by promoting chondrocyte proliferation and inhibiting apoptosis through the suppression of NF‐κB signalling, which is influenced by METTL3. Moreover, NEK7, a crucial regulatory protein of the NLRP3 inflammasome involved in pyroptosis, is affected by METTL3. Xiong et al. [59] revealed that lower METTL3 levels lead to m^6^A modifications on NEK7 mRNA, increasing NEK7 expression and subsequently influencing pyroptosis‐related proteins (NLRP3, ASC, caspase‐1, and GSDMD) and inflammatory cytokines (IL‐1β, IL‐18, IL‐6, IL‐10, and TNF‐α), thereby inhibiting chondrocyte pyroptosis and OA progression (Figure 1, Table 4).

#### 3.1.3 Extracellular Vehicles (EVs) Exert Their Effects Through the Suppression of METTL3 Activity

EVs derived from bone marrow stem cells (BMSCs) exhibit no significant adverse effects, making them viable in the treatment of various diseases, including OA. The therapeutic action of BMSC‐EVs in OA is intricately linked to the function of METTL3 in the apoptosis of OA cells. For instance, Cheng et al. [60] revealed that BMSC‐EVs downregulate METTL3 expression, which enhances the stability and expression of ACSL4 mRNA through m^6^A modification. The METTL3‐ACSL4 pathway is instrumental in promoting cell survival, reducing levels of Fe^2+^/ROS/MDA, elevating GSH levels, and diminishing the number of apoptotic cells. Importantly, the protective role of BMSC‐derived EVs in OA is associated with the METTL3‐ACSL4 pathway, which mitigates chondrocyte ferroptosis and ultimately slows OA progression [60]. Consistent with this, Zhou et al. [61] demonstrated that EVs from human umbilical cord mesenchymal stem cells (hucMSCs) retard OA progression, decrease osteophyte formation, upregulate COL2A1 and aggrecan expression critical for ECM synthesis, and downregulate ADAMTS5 and MMP13 expression, which are implicated in ECM degradation in OA murine models, by reducing the secretion of pro‐inflammatory mediators. Furthermore, EVs promote chondrocyte proliferation and migration while suppressing apoptosis. Additional mechanistic investigations revealed that miR‐1208 within EVs targets METTL3, leading to reduced NLRP3 mRNA methylation, thereby diminishing the release of inflammatory factors and averting OA progression [61]. Consequently, the protective effects of hucMSC‐EVs in mitigating OA progression are closely associated with the anti‐apoptotic and anti‐pyroptotic actions of METTL3, presenting a novel therapeutic avenue for clinical OA management (Figure 1, Table 4).

In summary, existing literature suggests that OA interventions predominantly target the inhibition of chondrocyte mortality induced by inflammatory responses, including apoptosis, autophagy, ferroptosis, and pyroptosis, all of which are significantly correlated with the downregulation of METTL3 and the mechanisms of METTL3‐mediated m^6^A modification. These insights indicate that the suppression of METTL3 expression is a pivotal strategy for OA treatment, warranting the exploration of specific METTL3 inhibitors from a clinical standpoint.

### 3.2 Targeting METTL3 Presents a Therapeutic Opportunity for OP Patients

The management of bone defects has emerged as a significant clinical challenge due to the impaired bone regenerative capacity observed in individuals with OP [62, 63]. Mesenchymal stem cells, including BMSCs and adipose‐derived stem cells (ASCs), demonstrate efficacy in promoting bone regeneration and are extensively utilised in regenerative medicine for OP treatment [64]. Research by Wu et al. [65] revealed a downregulation of METTL3 expression, correlating with diminished osteogenic potential in BMSCs from OP patients. Enhanced METTL3 expression in BMSCs stimulates osteogenic‐related factors and activates the Wnt signalling pathway, thereby facilitating the restoration of bone formation capabilities in OP individuals [65]. Furthermore, studies by Song et al. [66] and Luo et al. [67] indicated that both the osteogenic differentiation potential and METTL3 expression are markedly reduced in OP‐ASCs. The overexpression of METTL3 significantly improves the osteogenic differentiation of human ASCs in OP through the activation of the MAPK signalling pathway [67].

Collectively, these findings indicate that the upregulation of METTL3 enhances the osteogenic differentiation and migratory capabilities of BMSCs and ASCs, whereas silencing METTL3 diminishes osteogenic gene expression and exacerbates OP. Consequently, METTL3 emerges as a viable therapeutic target for addressing bone defects and promoting bone regeneration in OP patients, with strategies to augment METTL3 expression representing a promising avenue for OP treatment.

### 3.3 Implications of METTL3 in OS Treatment

METTL3 plays oncogenic roles in OS, inferring that downregulating METTL3 might be a therapeutic target for patients with OS. However, publications about targeting METTL3 for OS treatment are not yet available, which needs further exploration and validation. Building on these insights, future research should also explore the potential of combining METTL3 inhibition with existing chemotherapeutic agents. The synergistic effects of such combinations may enhance the cytotoxicity of conventional treatments while mitigating the development of resistance. For instance, studies could focus on how the modulation of m6A methylation by METTL3 impacts the sensitivity of OS cells to doxorubicin or cisplatin, both of which are standard treatments in OS management. Furthermore, exploring the role of METTL3 in the regulation of non‐coding RNAs, such as microRNAs and long non‐coding RNAs, could provide crucial insights into its function in OS. These non‐coding RNAs are increasingly recognised for their roles in cancer progression and treatment response. Investigating the regulatory networks involving METTL3 and these non‐coding RNAs could unveil new biomarkers for prognosis and treatment efficacy.

## 4 Discussion

The m^6^A modification of RNA represents a dynamic and reversible post‐transcriptional mechanism that can influence RNA processing, splicing, export, degradation, and translation through the activities of “writers,” “erasers,” and “readers.” Current research indicates that METTL3, the key catalytic enzyme involved in m6A modification, plays significant roles in various bone pathologies, including OA, OP, and OS. However, there is a notable lack of specific reviews that elucidate the relationship between METTL3 and these conditions, leaving a gap in the understanding of its functions, mechanisms, and potential therapeutic applications. Consequently, this review thoroughly examines the contributions of METTL3 to OA, OP, and OS, aiming to provide a detailed interpretation and address this gap.

The findings of this review indicate that METTL3 acts as a risk factor for these diseases by mediating m^6^A modifications on both mRNA and non‐coding RNAs through various mechanisms. The functional mechanisms of METTL3 in these disorders involve the regulation of several critical cellular processes, including inflammatory cascades, ECM degradation, mitochondrial dysfunction, cell cycle regulation, proliferation, migration, apoptosis, pyroptosis, senescence, autophagy, ferroptosis, and reprogramming of aerobic glycolysis.

Specifically, elevated levels of METTL3 are associated with the onset and progression of OA by promoting the release of inflammatory cytokines, facilitating ECM degradation, and inducing chondrocyte apoptosis, thereby serving as a potential biomarker for OA diagnosis. Conversely, downregulation of METTL3 expression appears to have a protective effect in OA. Additionally, reduced METTL3 levels result in differentiation imbalances in BMSCs, contributing to the pathogenesis of human OP. Thus, we propose that increasing METTL3 expression may represent a viable therapeutic strategy for OP. Furthermore, this review highlights that aberrantly elevated METTL3 levels are prevalent in OS tissues and cells, where it enhances tumour cell proliferation, migration, and invasion while simultaneously inhibiting apoptosis, either by acting as an oncogene or by modulating oncogenic pathways indirectly. In conclusion, METTL3 expression exhibits distinct variations across OA, OP, and OS. Therefore, it is essential to evaluate both disease status and expression trends when considering METTL3 as a diagnostic or prognostic biomarker and therapeutic target in these disorders.

Nonetheless, limitations persist in fully elucidating the mechanisms by which METTL3 operates in bone diseases. For instance, despite the common observation of elevated METTL3 levels in OA, research by Sang et al. [68] has shown that METTL3 expression is diminished in clinical OA samples and reduced in chondrocytes treated with IL‐1β. Thus, the role of METTL3 in OA necessitates further preclinical investigation. Additionally, while some studies propose that targeting METTL3 or its associated signalling pathways could be beneficial for OA and OP, more extensive clinical data are required to substantiate this hypothesis. Moreover, the intricate relationship between METTL3 and DNA methylation in the context of bone and joint diseases warrants a deeper exploration. Recent literature has highlighted that DNA methylation, a key epigenetic modification, plays a pivotal role in regulating gene expression and cellular function in osteoblasts and chondrocytes [69, 70]. Specifically, studies have indicated that aberrant DNA methylation patterns can lead to the dysregulation of genes involved in bone remodelling and cartilage integrity, contributing to the pathogenesis of OA and OP [69, 70]. The interplay between METTL3 and DNA methylation may provide critical insights into the aetiology of these conditions. For instance, METTL3 is known to facilitate m^6^A methylation of RNA, influencing the stability and translation of mRNAs encoding proteins essential for bone homeostasis. However, its influence on DNA methylation processes, particularly through interactions with DNA methyltransferases or other epigenetic modifiers, remains underexplored. Given that DNA methylation can directly affect the transcriptional landscape of chondrocytes and osteoblasts, understanding how METTL3 modulates these modifications could unveil novel therapeutic targets. Furthermore, the potential for METTL3 to act as a regulatory hub linking RNA and DNA methylation pathways highlights the necessity for comprehensive studies that assess both mechanisms concurrently. For instance, examining how the modulation of METTL3 expression affects the methylation status of specific genes implicated in OA and OP could yield valuable insights into disease mechanisms. This could involve employing advanced genomic technologies, such as methylation sequencing and RNA‐Seq, to elucidate the broader epigenetic landscape influenced by METTL3. In addition, the observed discrepancies in METTL3 expression levels across different studies necessitate a critical evaluation of the methodologies used to assess its activity. Factors such as sample source, disease stage, and the microenvironment can significantly influence METTL3 expression and its downstream effects. Therefore, establishing standardised protocols for measuring METTL3 and its associated pathways in clinical samples is essential for drawing reliable conclusions about its role in bone diseases. Moreover, therapeutic strategies aimed at modulating METTL3 activity could offer innovative approaches to managing OA and OP. The development of small molecules or RNA‐based therapies that specifically target METTL3 or its downstream signalling cascades could potentially restore normal methylation patterns and improve cellular function in diseased tissues. Such interventions would benefit from rigorous preclinical testing to evaluate their safety and efficacy before progressing to clinical trials. In summary, while the current body of research sheds light on the potential roles of METTL3 in bone and joint diseases, significant gaps remain in our understanding of its mechanisms of action, particularly concerning DNA methylation. Future investigations must prioritise elucidating the multifaceted functions of METTL3 in the context of epigenetic regulation, as this knowledge could pave the way for novel diagnostic and therapeutic strategies tailored to combat the debilitating effects of OA and OP.

Furthermore, research into targeting METTL3 for OS treatment is limited, necessitating further exploration to clarify these connections. To further elucidate the implications of METTL3 dysregulation in OS, it is crucial to delve deeper into the cellular microenvironment and the heterogeneity observed within OS tissues. The single‐cell RNA sequencing study provides a comprehensive landscape of the various cell types present in OS [71]. These findings underscore the complexity of the tumour microenvironment, revealing that OS is not merely a homogeneous mass of malignant cells but a dynamic ecosystem consisting of tumour cells, immune cells, stromal cells, and possibly other progenitor cells. The distinct subtypes of OS, characterised by their unique histopathological patterns and clinical behaviours, further complicate our understanding of METTL3's role. For instance, conventional high‐grade OS, which is the most prevalent form, often exhibits significant METTL3 overexpression, correlating with aggressive tumour features and poor prognosis. In contrast, low‐grade OS subtypes may not exhibit the same level of METTL3 upregulation, suggesting that the role of METTL3 could vary significantly depending on the histological subtype. This variation indicates that therapeutic strategies targeting METTL3 may need to be tailored according to the specific subtype of OS, taking into account the underlying biological differences. Moreover, the interaction of METTL3 with various oncogenic pathways cannot be overlooked. Its influence on mRNA methylation affects gene expression regulation, which is pivotal in cancer progression. For example, METTL3‐mediated mRNA modifications may enhance the translation of oncogenic factors while simultaneously repressing tumour suppressor genes. This dual role positions METTL3 as a central player in the post‐transcriptional regulation of genes that dictate cellular behaviour in OS. Understanding these molecular interactions offers an avenue for identifying potential biomarkers that could predict response to METTL3‐targeted therapies. It is also essential to consider the immune landscape within OS tissues. The presence of immune cells, particularly tumour‐associated macrophages and lymphocytes, can significantly influence tumour progression and response to treatment. METTL3 can modulate the immune response in various cancers, and similar mechanisms may be at play in OS. By examining the interplay between METTL3 levels and immune cell infiltration in different OS subtypes, we may uncover novel therapeutic strategies that not only target METTL3 but also enhance anti‐tumour immunity. In light of these discussions, future research should prioritise elucidating the precise mechanisms by which METTL3 contributes to the tumour microenvironment in OS. Investigating the interplay between METTL3 expression and the presence of various immune cell types could yield insights into the potential for combination therapies that harness both direct tumour targeting and immunomodulatory approaches. In summary, while the current literature elucidates the significant role of METTL3 in OS progression, a more nuanced understanding of its function across different tumour subtypes and its interactions within the tumour microenvironment is essential. This multifaceted approach will be instrumental in developing targeted therapies that could improve outcomes for patients afflicted with this aggressive bone malignancy.

In conclusion, this review provides a comprehensive interpretation of the relationships involving METTL3 and suggests potential applications. It also addresses current limitations and calls for further research. Notably, this review bridges the existing knowledge gap regarding METTL3 in bone diseases, serving as a valuable resource for researchers pursuing fundamental studies and the development of innovative clinical therapies.

## Author Contributions

**Dongqiong Xiao:** conceptualization (lead), writing – original draft (lead). **Deshuang Zhang:** conceptualization (equal), writing – original draft (equal). **Yi Qu:** funding acquisition (equal), supervision (equal). **Xiaojuan Su:** writing – review and editing (lead).

## Ethics Statement

The authors have nothing to report.

## Consent

The authors have nothing to report.

## Conflicts of Interest

The authors declare no conflicts of interest.

## Data Availability

The authors have nothing to report.
